# Development of the Strengths, Skills, and Goals Matrix: a tool for facilitating strengths-based adolescent and young adult engagement in research

**DOI:** 10.1186/s40900-023-00502-w

**Published:** 2023-10-04

**Authors:** Brooke Allemang, Megan Patton, Katelyn Greer, Karina Pintson, Marcela Farias, Keighley Schofield, Susan Samuel, Scott B. Patten, Kathleen C. Sitter, Gina Dimitropoulos

**Affiliations:** 1 Faculty of Social Work, University of Calgary, MacKimmie Tower, 400-B3, 2500 University Drive, NW, Calgary, AB T2N 1N4 Canada; 2 Department of Pediatrics, Cumming School of Medicine, University of Calgary, 3300 Hospital Drive NW, Calgary, AB T2N 4N1 Canada; 3 Mathison Centre for Mental Health Research & Education, University of Calgary, 3280 Hospital Drive NW, Calgary, AB T2N 4Z6 Canada; 4 Department of Psychiatry, Cumming School of Medicine, University of Calgary, 3300 Hospital Drive NW, Calgary, AB T2N 4N1 Canada; 5 Child Health Evaluative Sciences, SickKids Research Institute, 686 Bay Street, ON M5G0A4 Toronto, Canada

**Keywords:** Adolescents and young adults, Graduate-level research, Patient engagement, Patient-oriented research, Peer mentorship

## Abstract

**Background:**

The involvement of adolescents and young adults (AYAs) with lived experience of health and mental health conditions as partners in research is increasing given the prominence of participatory approaches to research, including patient-oriented research (POR). Much of the relevant research is conducted by graduate students. While guiding AYA engagement frameworks and models exist, the processes of partnering with AYAs in patient-oriented graduate-level research projects have not been well established. Co-developed tools and practices are required to support strengths-based, developmentally appropriate AYA-graduate student partnerships.

**Objectives:**

The objectives of this commentary are: (1) to share the processes of partnership between a graduate student and five Young Adult Research Partners (YARP), (2) to describe the co-design and implementation of the Strengths, Skills, and Goals Matrix (SSGM), a tool for facilitating strengths-based AYA engagement in research, and (3) to outline considerations for applying this tool across a variety of research contexts with patient partners.

**Main body:**

Within the YARP-graduate student partnership, the SSGM offered extensive benefits, including tangible skill development, peer mentorship, and rapport building among all members. This tool offers strategies for strengths-based engagement practices which emphasize AYAs’ preferences and goals throughout POR projects. Practical recommendations and considerations for applying the SSGM within graduate-level research and beyond are described, including the importance of connecting AYAs’ current (and desired) skills to specific tasks within the research project and resulting outputs.

**Conclusions:**

The SSGM has possible relevance in a variety of settings given its broadly applicable structure. Future research could explore the adaptation, application, and evaluation of the SSGM across research contexts to determine its feasibility and ease of implementation.

**Patient or public contribution:**

This article was conceived of and co-authored by five young adult research partners. The YARP co-designed the SSGM presented in this article, the figures, and substantially contributed to the preparation of the article.

## Abstract (plain language summary)

The involvement of adolescents and young adults (AYAs) with lived experience of health and mental health conditions as partners in research is becoming more common in student research projects. Though guidelines for engaging AYAs as partners in research exist, the steps for developing meaningful AYA-student partnerships are not clearly defined. These partnerships require tools and practices that are co-developed by AYAs and students to be successful. In this commentary, we share the details of a partnership between a graduate student and five AYA research partners. Next, we describe how we developed the Strengths, Skills, and Goals Matrix (SSGM), a tool for supporting AYA engagement in research which emphasizes the capabilities and goals of each AYA partner. Finally, we outline suggestions for patient partners and research teams interested in using this tool in different settings. Within our research partnership, the SSGM helped all members build relationships, develop skills, and share their skills with one another. The SSGM focuses on the strengths of each person and allows patient partners to determine their own goals for engaging in research. Future research should explore how the SSGM works for different types of research teams or projects.

## Introduction

Engaging adolescents and young adults (AYAs) in the design, conceptualization, and conduct of health and mental health research is gaining popularity with the prominence of participatory research approaches like patient-oriented research (POR) [1]. The involvement of AYAs with lived experience in health and mental health systems generates research projects that are responsive to the needs of AYAs themselves and findings that can be more readily implemented into clinical practice [2]. In addition, successful AYA-adult research partnerships produce positive outcomes for researchers and AYAs alike, including capacity building, reciprocal learning, and mutual growth [3]. Frameworks and models for youth engagement in research (e.g., McCain Model [3]) and tools to operationalize patient partnership in research (e.g., Involvement Matrix [4]) are available to support researchers in understanding how to involve AYAs in research projects. The principles of valuing experiential knowledge, engaging in mutual learning in order that AYA do not feel patronized or tokenized, and respecting the diversity of opinions offered by AYA are highlighted as critical to fostering an environment of meaningful AYA engagement within the literature [5]. Additional considerations for establishing and implementing AYA-adult partnerships in research include flexible engagement practices, mentorship opportunities, and authentic decision-making to ensure AYA feel valued and appropriately acknowledged for their contributions [3].

While patient engagement tools and frameworks for establishing meaningful partnerships exist, few studies have reported outcomes (e.g., skill development, capacity building) resulting from implementing AYA engagement tools. In addition, literature outlining the specific processes for actively engaging AYAs with lived experience in graduate-level research projects is limited. For the purposes of this article, graduate-level research is defined as research conducted in any faculty/department that is led by a masters or doctoral student. Unique considerations for this work are required given the timelines and expectations of graduate students and the developmental needs of AYA partners. This commentary aims to: (1) share the processes of partnership between a graduate student and five Young Adult Research Partners (YARP), (2) describe a co-designed, strengths-based tool which supported skill development within this partnership, and (3) outline considerations for applying this tool in other research contexts. It builds on the patient engagement literature by documenting specific processes, learnings, and reflections on the partnership and tool from the perspectives of AYA research partners themselves, with implications for patient partnership beyond graduate-level research.

## Development of the young adult research partnership

To bring the crucial voices of AYAs to a doctoral-level social work research project focused on pediatric-adult transitions for AYAs with health and mental health conditions, a YARP group was formed in March 2021. The overarching study adopted a patient-oriented, sequential explanatory mixed methods design to examine the transition readiness of AYAs with co-occurring chronic health and mental health conditions [6, 7]. Using a combination of transition readiness scores (quantitative data) and interviews with AYA with co-occurring conditions (qualitative data), this study aimed to explore the breadth and depth of AYAs’ experiences preparing for healthcare transitions while managing simultaneous physical and mental health concerns [6]. The methods and results of the overarching study within which the YARP was embedded have been previously published [7–9] and details about the YARP’s composition and roles in the project are described elsewhere [6–9]. Briefly, the YARP consisted of five female-identified young adults aged 18–30 across Canada with lived experience in the health system, mental health system, or both. The engagement of the YARP brought the perspectives of young adults to the study, ensuring the methods, analytic approaches, and products aligned with their needs. Members were recruited through existing patient engagement networks and youth advisory councils using a targeted email strategy. Monthly YARP meetings were organized and co-facilitated by the graduate student between March 2021 and September 2022, aligning with the timeline of the doctoral program.

During initial YARP meetings, the graduate student provided training to the YARP about the study objectives, design, and data collection tools, offered updates on the progress of the project, and presented quantitative variables and findings to YARP for input/interpretation. Throughout the partnership, YARP members began adopting more active roles in meetings by co-facilitating discussions, suggesting agenda items, and presenting their own ideas for knowledge translation outputs, for example. As the monthly meetings became more collaborative in nature, the graduate student and the YARP sought to implement tools and processes for identifying specific tasks for YARP members that aligned with their interests and goals (e.g., developing study recruitment materials, interpreting qualitative findings). This patient-oriented, mixed methods study received ethical approval from the Conjoint Health Research Ethics Board at the University of Calgary (REB#20-1928).

## Processes of partnering & meeting structure

**Fig. 1:**
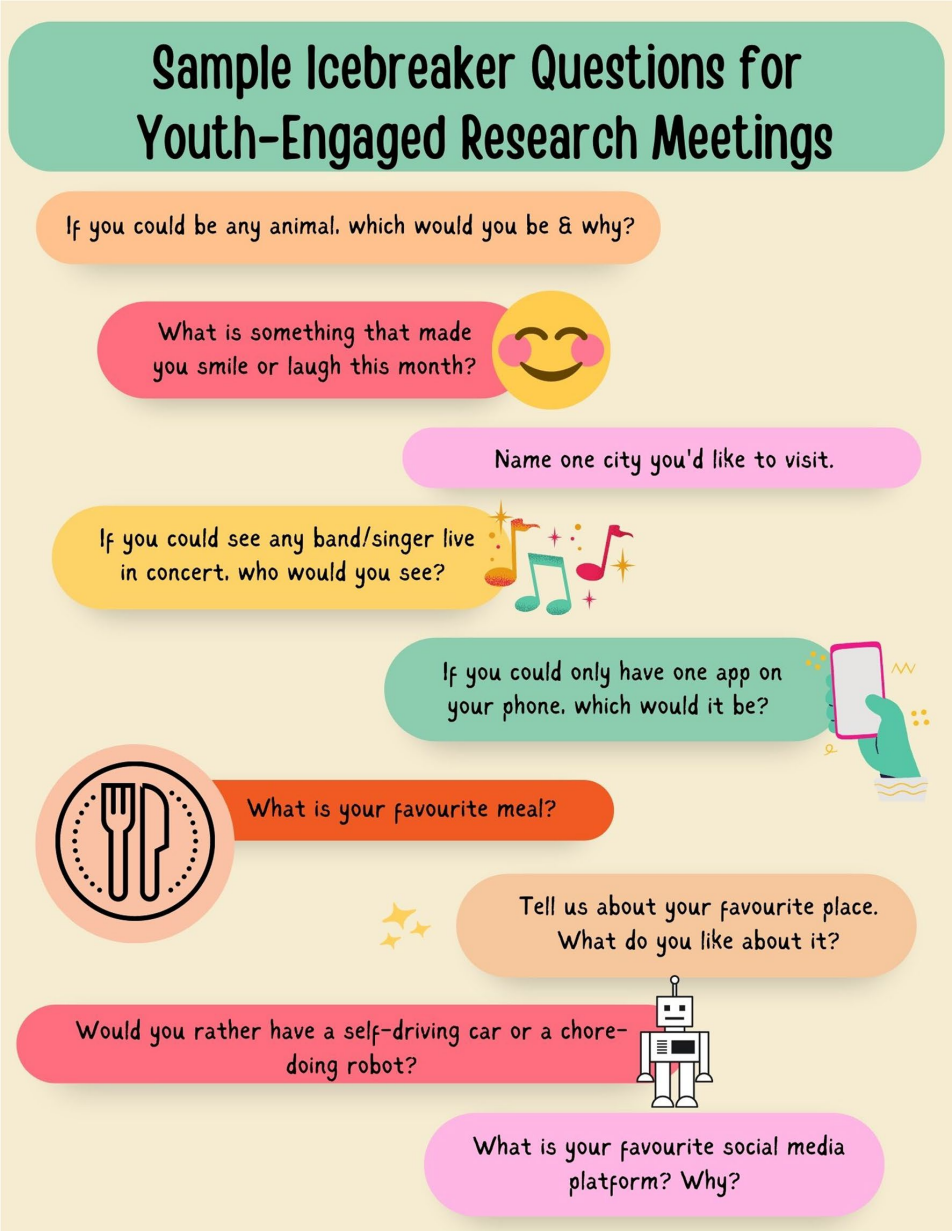
Sample Icebreaker Questions for Youth-Engaged Research Meetings

Within this project, the processes of partnering and group structure were co-developed, allowing the graduate student and YARP to share responsibility for decision making. Several strategies were employed to facilitate a developmentally appropriate approach to engagement within this partnership, including a focus on peer-to-peer connections, the use of technology, flexible meeting times to accommodate school/work/volunteer schedules, and choice in how the YARP were compensated and acknowledged. For the duration of the study, the YARP met monthly via the Zoom platform. A week prior to each meeting, all materials were emailed out to YARP members (e.g., agenda, slide deck, activities) by the graduate student. Meetings began with an icebreaker and check-in question and ended with a check-out question (see Fig. 1 for examples) which was led by a meeting co-chair; a role which rotated between YARP members. Check-in questions offered the YARP and the graduate student the chance to share fun facts about themselves with the group to assist with relationship-building, while check-out questions supported members in reflecting on the process of each meeting, key takeaways, and next steps. The YARP reported this flexible structure created an engaging environment that allowed for rapport to be built amongst the group and created comfortability and trust amongst members.

A terms of reference document was co-designed by the YARP and the graduate student to set the intentions and expectations of the group. This process included communicating clear descriptions of roles, responsibilities, time commitment, and compensation which created accountability and transparency from the outset. Collaborating on the development of the group’s terms of reference reportedly made the process of engagement less daunting for the YARP, as every member was involved in defining the scope of their own engagement in the project.

This project utilized technology to allow all YARP the opportunity to participate and contribute their ideas. Notably, the group created a shared drive for study materials, giving YARP members autonomy over how and when they could work on study tasks. This shared drive also increased opportunities for collaboration with each member of the team, as YARP members uploaded various educational resources to share with each other (e.g., presentation resources, research materials/tools, learning opportunities). As a group, the YARP decided to utilize an online messaging application for communication about project-related tasks rather than relying solely on email. This gave the team a casual way to connect with each other which helped facilitate peer-to-peer connections. In addition, the online messaging application was beneficial for celebrating each member's successes (e.g., awards, conference co-presentations) and sharing updates on available learning opportunities (e.g., webinars, conferences).

At the outset of the partnership, each YARP filled out the Involvement Matrix [4], a tool that provided members the opportunity to reflect on and define their desired roles and level of engagement in the graduate-level research project. Prior to completing this tool, the YARP were given a list of possible tasks and projects they could partner in by the graduate student and were encouraged to brainstorm other ways they wanted to be involved. While completing the Involvement Matrix [4], the YARP noticed the variety of strengths and skills each member possessed. The diverse skill sets amongst the group prompted the YARP to collectively design a tool that would encourage further skill development and peer mentorship opportunities.

## Co-development of the Strengths, Skills, and Goals Matrix (SSGM)

In establishing the YARP’s processes of partnership, an environmental scan was conducted by the graduate student to identify tools and resources to support task and role delineation throughout the research process. Several established patient engagement frameworks and best practice guidelines were reviewed (e.g., Canadian Institutes of Health Research Patient Engagement Framework [1], McCain Model [3], INNOVATE Guidebook [10]), including POR onboarding guides [11], training resources [12], growth grids related to youth skill development [13], and questionnaires regarding engagement practices [14]. Commonly encountered elements within these resources included the criticality of supporting patient partners to clearly identify their goals for engaging in research, encouraging all stakeholders to reflect on the competencies they were bringing into the project, and inviting members to consider which capacities they hoped to develop through the partnership. Existing resources typically took the form of worksheets or reflective activities administered at the outset of partnership, however, few tools were available specific to graduate-level research. This perceived gap in the peer-reviewed and grey literature informed the group’s decision to consider novel and context-specific practices for partnering in research.

The graduate student brought the concept of using tools to identify the strengths and interests of YARP members to the group. All YARP members expressed an interest in participating in reflexive activities (i.e., opportunities to consider their competencies, values, and positionality in reference to the research topic and to reflect on future goals) to promote their growth and provide direction in skill development within the confines of the time-limited graduate research project. Though there were elements of existing resources that aligned with the YARP’s goals (e.g., a focus on identifying desired skills), it was determined amongst the group that a *collaborative tool* for documenting each member’s strengths, skills, goals for engagement, and tangible outputs be developed to support transparency and within group skills-sharing. As such, a project-specific Strengths, Skills, and Goals Matrix (SSGM) was created (See Table 1 for the SSGM with example rows from YARP members included).

**Table 1:** Strengths, Skills, and Goals Matrix (SSGM) template

Name	Current skills or strengths (e.g., public speaking, social media, research experience)	Skills or strengths you would like to develop	Peer mentorship/ matching plan	Contact information & communication preferences (e.g., email address, phone number; include if interested in individual/group communication)	Outcomes or progress with skill development (e.g., list of presentations given, understanding of Instagram insights)
Example: YARP 1	*Research*: Quantitative- data analyses, analyzing and organizing data, and interpreting/presenting results. Qualitative- conducting lit reviews, scoping/systematic reviews, recruitment, qualitative data analysis and coding *Social media*: research recruitment and knowledge translation, management of organization social media accounts, content creation	I would like to build my public speaking skills. Public speaking is a challenge, I get so nervous so getting a bit more comfortable with that would be amazing	YARP 2 is assisting with public speaking, I am assisting them with academic writing. We will meet via Zoom for practice presentations and to discuss processes related to academic writing	Whatsapp	-7 co-presentations -Improvement in public speaking skills reflected in presentation marks at school -Created the study logo -Developed 3 videos which were shared through project social media accounts as knowledge translation outputs
Example: YARP 2	*Communication*: public speaking, social media, peer support, mediation, professionalism, constructive criticism, project management, outreach, trauma informed *Business*: minor management, budgeting, finances, funding, sales, marketing, organization	*Education*—Looking for advice on funding education (scholarships etc.) looking to learn more about the way the educational system is structured and how to better navigate it, standards or practice in education. Interested in learning more about the academic writing process *Data*—very interested in learning more about how to read, organize, present data, and collect data	YARP 1 is assisting with developing academic writing skills, I am assisting them with public speaking. We will meet via Zoom to walk through writing processes	Whatsapp	-Improved confidence and skill set in academic writing -Co-authored three articles with the graduate student & YARP -Better understanding of how to present data -Enhanced knowledge of mixed methods research and integrating quantitative and qualitative findings -Presented findings related to this project at 3 conferences

The overarching study was guided by the theory of positive youth development [15], thus, focusing on the resiliencies of the YARP was paramount throughout our partnership. The SSGM differs from existing patient engagement tools in that it is underpinned by a strengths-based approach, wherein an individual’s character strengths and skills are celebrated [16], and incorporates opportunities for peer mentorship and support. Another unique feature of the SSGM as applied to the current project was the collaborative nature of the tool, whereby all YARP completed their own rows on the same version of the document to promote the generation and exchange of ideas. Lastly, including the final column to track concrete outcomes or skill acquisition allowed the YARP to reflect on their key learnings and outputs related to the project in a consistent location. These outcomes could then be included on resumes or in cover letters when YARP members were applying for other opportunities. The SSGM was particularly well-suited for graduate-level research, as it allowed both  the graduate student and the YARP to reflect on areas for growth and development given both parties were in the early stages of their research careers.

The SSGM is a tool consisting of a collaborative document with a table that includes information on each group member’s current and desired skills which is updated in real-time. Each YARP member and the graduate student filled in their own row of the collaborative SSGM document housed on a shared drive, which allowed them to view each other’s responses, and updated their row on an annual basis. The development of the SSGM originated from initial YARP meetings when considering how peer support models might benefit the group. These discussions highlighted the criticality of informational and relational support for YARP members when building new skills, the importance of peer-to-peer connections within the group, and the mutual benefits gleaned from assisting others in developing competencies. Conversations focused on peer mentorship emerged organically due to YARP members’ previous experiences with peer support and the group’s collective objective of capacity building.

Developmentally appropriate practices were incorporated into the design and implementation of the SSGM. For instance, YARP members voiced the importance of developing particular skills (e.g., qualitative analysis, academic writing) that would support their professional development given their life stage. Most YARP members were actively seeking employment or applying to post-secondary programs at the time of this project, thus, they expressed the value of tracking specific activities associated with the research partnership (e.g., presentations given, number of social media posts developed) in order to build their resumes and speak to their capacities in interviews. These reflections informed the addition of the “outcomes or progress with skill development” column.

When developing this tool, the YARP valued the concept of each member having autonomy over how much detail to include in the SSGM. After the first year of SSGM utilization, the tool was revisited and discussed amongst the group. At this point, the YARP added columns to support the ongoing partnership, including peer mentorship plans, contact information/communication preferences, and outcomes to track one’s progress over time. The "peer mentorship" column supported the intentional matching of YARP members based on desired skills, whereas the "contact information" column was included to allow YARP members to explicitly state their communication preferences. The "outcomes or progress with skill development" column offered a place to observe, reflect on, and redirect efforts if needed, and to visualize tangible outcomes from the previous year, in part due to the utilization of the SSGM. Skill development is a prominent aspect of graduate-level research, as graduate students are frequently engaging in activities which foster the solidification of new competencies they will apply throughout their training and future careers. As such, a sense of reciprocity was possible in this project, where the graduate student sought advice and learning opportunities from the YARP in a variety of areas.

The direct access to the SSGM, which was housed on a shared drive, provided the opportunity for all group members to review each other’s strengths and skills without going through a third party. This facilitated peer/sideways mentorship among the YARP in addition to mentorship by the graduate student. As a result, the ‘gatekeeping’ role of researchers commonly observed in health research was circumvented, creating a more casual and flexible environment which made communication (and reaching out) about skills and goals more comfortable for the YARP. This peer mentorship structure was suitable for the age range of the YARP and highly valued by all members. The reciprocal peer-to-peer learning environment evoked feelings of pride in the group’s work and allowed each member to feel both valued and valuable [17].

## Implementation of the SSGM within the YARP

**Fig. 2:**
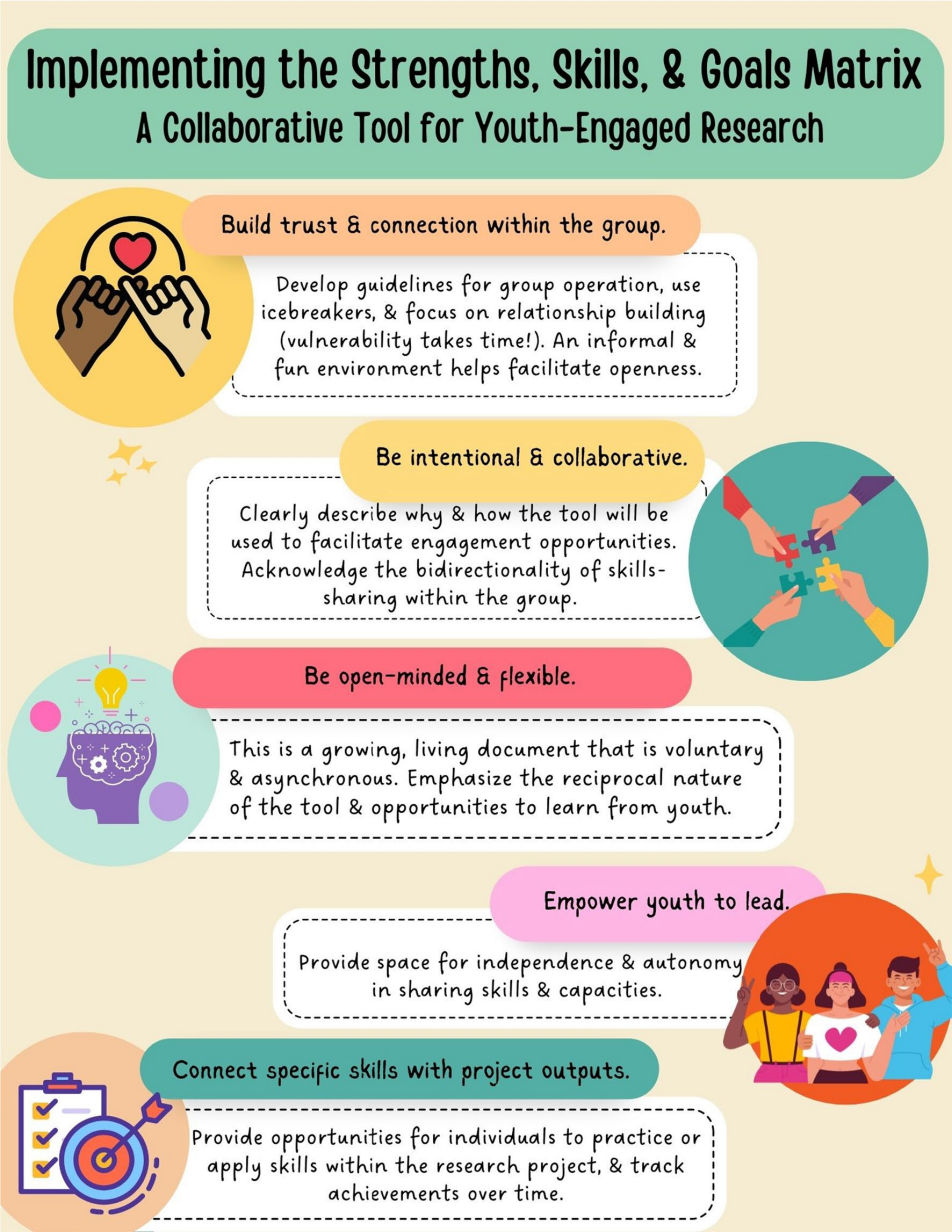
Implementing the Strengths, Skills, & Goals Matrix

The graduate student and the YARP learned useful lessons as they co-developed and implemented the SSGM within the partnership. We’ve summarized these learnings based on our experiences in Fig. 2. Key reflections included the importance of developing trust and rapport prior to completion, empowering young adults to determine how they would like to use the tool, and connecting AYAs’ current (and desired) skills to specific tasks within the research project and resulting outputs. In addition, approaching the adoption of this tool with a clear set of guiding values as agreed upon by the group, including openness to learning, authenticity, and reciprocity, was an important takeaway from our partnership. In this section, we describe these learnings with reference to how the SSGM was applied within the YARP, as well as strengths and challenges to implementation of the tool.

### Build trust and connection within the group

Trust and authenticity were paramount to establishing a meaningful AYA-researcher partnership in this project. Efforts were made to create connections between members (at and in between meetings) by allowing time for informal conversations about life outside of the research study, goals for the future, motivations for becoming involved in research, and icebreaker questions. These activities were incorporated into every team meeting throughout the partnership which helped to establish rapport and a sense of comfort in sharing strengths and hopes for future skill development using the SSGM.

### Be intentional and collaborative

Once the format of the SSGM tool was solidified within our project, members determined its completion would be optional, supporting the autonomy of each YARP member and the graduate student. Group members, including the graduate student, completed the SSGM outside of scheduled meetings via a collaborative document on the shared drive. Information that was entered into the SSGM was visible to all members immediately which provided an unforeseen benefit of additional ideas about what to include in one’s current or desired skills sections. Without the option of seeing other members’ input on the SSGM, members may not have considered including certain skills and strengths in their own sections. This collaborative process revealed “hidden” skills that were not immediately evident as relevant to the research project but were ultimately intentionally utilized (e.g., social media management). Furthermore, this process uncovered YARP members’ interests outside of the research project, further strengthening personal relationships among members. All information provided in the SSGM was self-reported, and YARP members were not expected to be experts in the subject matter or provide evidence of competency of their knowledge or skills.

### Be open-minded and flexible

Though the SSGM was optional, it was utilized by all group members (including the graduate student) and reported to be highly beneficial by the YARP. Given the YARP were involved in the graduate-level research project over an 18-month period, the tool was revisited and each member’s progress was discussed on an annual basis. The described growth in knowledge and skills was not limited to YARP members but was also described by the graduate student. The tool itself was a living document which could be modified by any member of the team throughout the partnership. For example, additional columns were included in year two of the project based on YARP members’ hopes for more intentionally tracking their progress and contributions to the study.

### Empower youth to lead

While the graduate student completed the SSGM, she was not directly involved in facilitating peer-to-peer connections between YARP members based on their responses on the SSGM. During the development of the SSGM, the YARP expressed an interest in taking ownership of connecting with peers based on their individual needs and preferences. If a task presented itself during the research project (e.g., conference co-presentation) involving a skill that a YARP member did not possess (e.g., confidence in public speaking), the YARP could refer to the SSGM themselves and connect with a member who identified that skill (e.g., public speaking) as being a strength. This self-directed process was beneficial, as it allowed the connections to be timely, authentic, informal, and comfortable for YARP members to discuss areas of growth without fear of judgment. Of note, YARP members had choice over whether they wanted to mentor or be mentored by another AYA based on their interests, availability, and motivations and this was not a requirement of the research partnership. If there were skills YARP members hoped to develop that did not align with other YARPs’ strengths, the graduate student sought out opportunities to provide education/support to enhance specific skills where possible (e.g., in mixed methods research or qualitative analysis).

The peer-to-peer mentorship often occurred in one-on-one meetings outside of regularly scheduled YARP meetings using a preferred method of communication including online messaging platforms or via video-conference. For instance, two YARP members met via Zoom to discuss strategies for managing stress during public speaking engagements and to practice reviewing slides in preparation for conference co-presentations related to the project. In this example, the mentor was able to provide tips and tricks that aided in their confidence with public speaking. The two YARP members held mock presentations to practice these skills in a low-pressure environment before implementing them in academic co-presentations.

During these peer-initiated meetings, the YARP member offering mentorship was not required to be an expert in the skill or strength; rather they offered knowledge based on their experience with that particular skill. This mutual understanding made the meetings less formal and prescriptive, which led to positive outcomes for both parties. The mentor gained experience and confidence offering their expertise, and the mentee made progress towards a skill they were looking to develop in a safe and encouraging environment. This dynamic was also beneficial because the advice was delivered by a peer, making it relatable to the mentee given their closeness in age and life stage. The learning could be reciprocal, with the roles of mentor and mentee being switched based on the skill or the strength being developed. In addition, the YARP noted the style of leadership and interaction with the graduate student to be particularly productive since they were of similar age. If applicable, members would upload resources to the shared drive to aid in mentoring other YARP.

### Connect specific skills with project outputs

YARP members reflected on how the SSGM assisted them in organizing and tracking the acquisition of specific competencies related to research, professional development, and social skills. YARPs’ reflections on the tool, and the partnership more broadly, were obtained through annual team discussions and optional memos. At the request of the YARP, the graduate student facilitated informal group discussions on an annual basis about key learnings, perceptions of engagement strategies (including tools used), meeting structure, and hopes for future directions. Guiding questions were sent to YARP members in advance of these annual discussions to allow them to prepare. In addition, the graduate student developed a memo template for YARP members which included prompts and headings that could be used to document their experiences of the partnership. Memos were optional and could be completed anonymously at any point during the partnership. YARP members who were interested in sharing their reflections on the tool and engagement process sent their memos to the graduate student for review. The memos and group discussions contained striking examples of members getting to practice and apply specific skills outlined in the SSGM (e.g., delivering oral presentations) and the subsequent outcomes (e.g., improved confidence in public speaking skills, greater comfort expressing thoughts in team meetings).

The ability to articulate their hopes and goals for engagement in research and subsequently monitor their progress over time were described as key benefits of the SSGM by the YARP. The YARP felt that having autonomy over their involvement also supported sustained interest in the project. The skills all members, including the graduate student, acquired through the project are transferable to a variety of other contexts, both personally and professionally. The YARP members shared that the relationships, knowledge, and skills gained as a result of the SSGM extended beyond the graduate student's thesis, including lasting relationships with each other, understanding of mixed methods research, stronger writing skills, confidence in peer mentorship, and presentation skills. For example, one YARP who prioritized public speaking skill development shared that through this project, they noticeably increased their ability to deliver presentations at school. An outcome they shared with the group after receiving peer mentorship and delivering several co-presentations was a high mark on an assigned presentation with excellent feedback relating to their ability to seamlessly speak to the class. This YARP had previously found class presentations to be very challenging, resulting in low marks and significant feedback. Another YARP member who is involved in several research initiatives now advocates for using the SSGM at the outset of all projects to support group-based and individual goal setting.

### Strengths and challenges to implementation

The co-designed SSGM offered solutions to common barriers in AYA engagement in our partnership. Although researchers possess a variety of skills relevant to patient-engaged research, time with leadership is often a limited resource. Researchers may lack adequate time to build capacity with AYA research partners. This tool provided a platform to acknowledge the unique skills and strengths that every AYA brought to the research team, including specific competencies that researchers may not possess. As such, it utilized a strengths-based approach [16] to AYA engagement underpinned by the theory of positive youth development [15], wherein the capacities of AYAs are honoured and acknowledged. The decreased power dynamic between peers allowed AYAs to be more comfortable while making progress towards desired skills (e.g., qualitative data analysis, social media management, public speaking), while creating strong relationships with each other.

While implementation of the SSGM within this project was quite seamless, likely due to the high levels of motivation and cohesion among the YARP, the process of developing and utilizing the tool was not without its challenges. In the initial stages of the research partnership, group guidelines regarding communication processes and timelines for task completion were still being solidified. This meant that some members contributed their responses to the tool as soon as it was co-developed, while others required prompting and reminders to do so. To address this challenge, the graduate student added “discussion of the SSGM” to the monthly meeting agenda once its format was agreed upon by the group and set deadlines for completion. The YARP and the graduate student were invited to share their own rows of the SSGM with the group at meetings which aided in creating a sense of accountability to the group and connection to other members. The opportunity for sharing and discussion also assisted YARP members in considering alignments between their existing strengths and those their peers hoped to develop. In addition, YARP who engaged in peer-to-peer mentorship outside of monthly meetings elected to share their progress with skill development and their processes of supporting each other with other members. These updates from YARP members were added to monthly meeting agendas (when requested) and served to encourage other members to consider engaging in sideways mentorship with their peers. Transparent discussions about the purpose of the tool, timelines for completion, the roles of group members, and expectations surrounding sharing progress with peers were held in order to mitigate challenges with implementation.

## Considerations for applying the SSGM

The format of the SSGM gives purpose, meaning, and direction to patient-engaged research projects and allows for its possible application to different settings, institutions, or groups. The SSGM was developed for the purposes of a time-limited graduate-level research project during the COVID-19 pandemic, therefore, the application and implementation to other contexts (e.g., larger research projects, education, healthcare, advocacy groups) should be adapted based on the group’s local needs, resources, and goals. For instance, patient partners with limited knowledge of or experience with peer support may benefit from training on establishing appropriate mentorship plans, building rapport, setting boundaries, and self-care prior to the tool’s use. Such training could be led by patient partners themselves to promote the values of sideways mentorship or research teams could partner with community organizations offering peer support education and training programs.

**Fig. 3:**
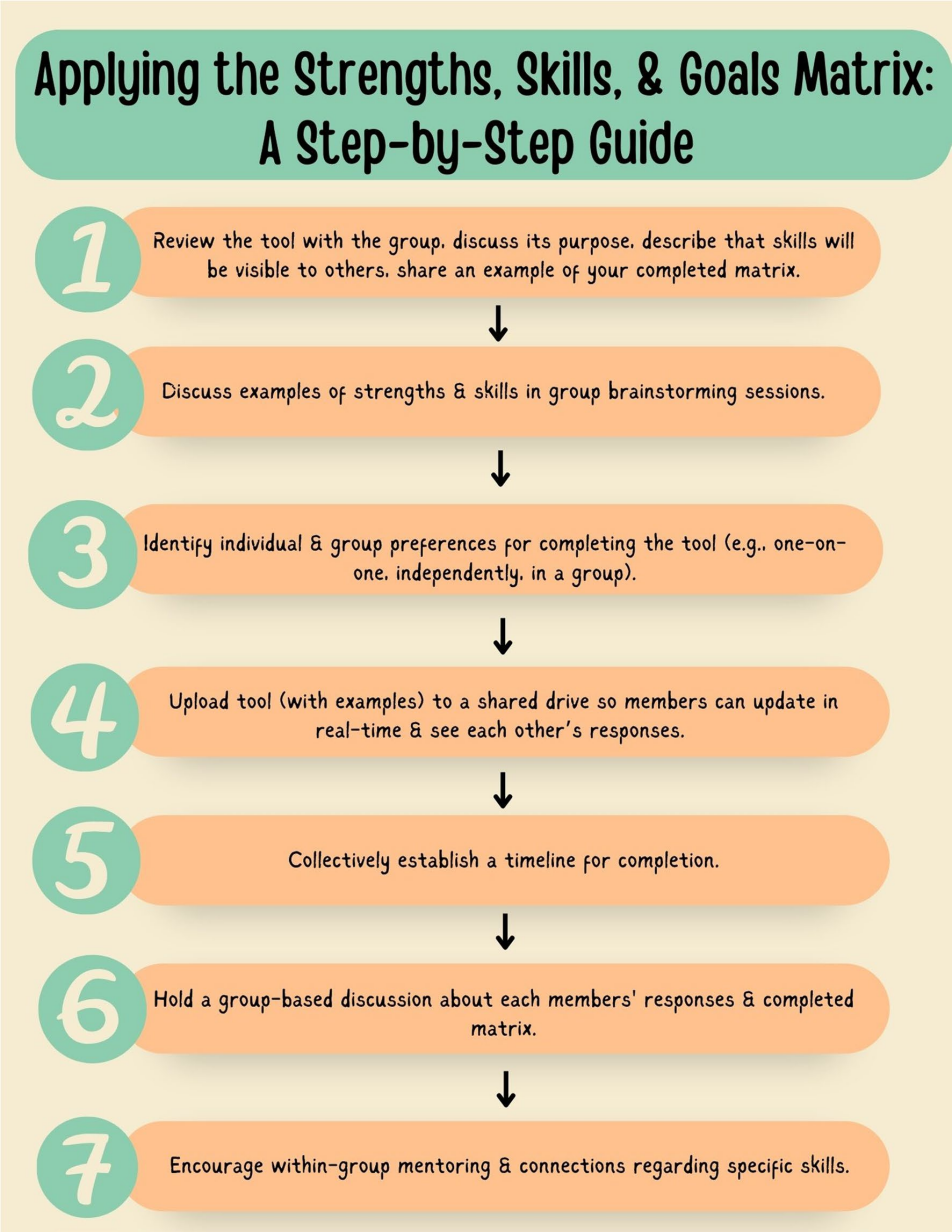
Applying the Strengths, Skills, & Goals Matrix: A Step-by-Step Guide

Our guide to application of the SSGM (Fig. 3) highlights the step-by-step process of introducing and implementing the tool based on our collective experiences. The process involves clearly describing the SSGM and its purpose, providing examples of possible strengths or skills relevant to the project, identifying the group’s preferences for completion (e.g., independently vs. in a group), and organizing large group discussions to promote skill-sharing, group connections, and within-group mentoring. Uploading the SSGM to a shared drive to allow the document to grow and evolve over the duration of a project was identified by the YARP as another important consideration for its application. Collectively deciding on a timeline for completion was a key component in the success of our implementation. This included deciding when the tool should be independently completed and when the group would share responses. YARP members found it beneficial to share the positive outcomes of their peer mentorship activities in group meetings as it encouraged further mentoring and connection building.

Of note, the SSGM was co-designed and implemented by a group of five AYA partners and a graduate student who collaborated on a research project over an 18-month period. As such, some features of the tool align with the developmental needs of AYA (e.g., tracking outcomes to include on resumes, interest in connecting with peers) and may be suited for small, highly engaged groups who meet on a consistent basis (e.g., monthly). When applying this tool within smaller/larger teams, with partners from different developmental stages/educational backgrounds, or on projects with varying engagement structures (e.g., monthly vs. annual meetings), tailoring may be required. For instance, large teams could be broken up into smaller groups to complete the SSGM to allow adequate time for each member to share their skills and strengths verbally and seek advice from the team about how to build desired competencies. Researchers could become more involved in initiating peer-to-peer matching in groups which meet less frequently. Children or youth engaged as patient partners may be more comfortable expressing their ideas verbally and having a notetaker transcribe their reflections onto the SSGM. In projects involving long-term patient engagement, the SSGM could be formally revisited at regular intervals as determined by the group (e.g., bi-annually or annually) to support transparency and outcome tracking. Ultimately, the SSGM provides a strengths-based framework for initiating group discussions about current skills, goals, and desired outcomes and can be applied flexibly based on the context within which it is being implemented. Future research should aim to examine the relevance and transferability of this tool to POR projects with teams of varying sizes, compositions, ages, and life stages.

## Conclusions

This commentary described the processes of partnering with AYAs in a graduate-level research project and the co-development and application of a strengths-based tool for facilitating skill development within this partnership. The intentional use of the SSGM within the YARP supported all members (including the graduate student) in articulating their strengths, identifying possible tasks of interest within the research, and reflecting on desired areas of growth. The format of the SSGM, which was modifiable by all members in real-time, promoted YARP members’ autonomy over its completion and initiated within group skills-sharing. Approaching AYA engagement from a strengths-based perspective within this project yielded a wide range of benefits for all members, including a sense of ownership over project outputs, bolstering of young people’s capacities, recognition of skill development and pride in the group’s achievements. The value of tracking each individual’s milestones and progress was observed and celebrated and the SSGM offered a structured and organized format for doing so.

While the SSGM was conceived of in the context of a graduate student-AYA partnership, this tool could be used on teams with researchers at more advanced stages of their careers and patient partners of different ages and stages given its flexible nature, focus on relational aspects of partnership, and grounding in a strengths-based framework. Tools like the SSGM implore researchers to critically examine their engagement practices and adopt intentional approaches to partnership that align with the goals of patient partners. The SSGM has possible relevance for larger scale POR projects, advisory councils, and educational settings. Future research could explore the SSGM’s use, application, and adaptation across a variety of contexts to determine its feasibility, utility, and ease of implementation.

## Data Availability

Not applicable.

## Abbreviations

AYA: Adolescent and young adult
POR: Patient-oriented research
SSGM: Strengths, skills, and goals matrix
YARP: Young adult research partners
